# Investigating the Diversity and Host Range of Novel Parvoviruses from North American Ducks Using Epidemiology, Phylogenetics, Genome Structure, and Codon Usage Analysis

**DOI:** 10.3390/v13020193

**Published:** 2021-01-28

**Authors:** Marta Canuti, Joost T. P. Verhoeven, Hannah J. Munro, Sheena Roul, Davor Ojkic, Gregory J. Robertson, Hugh G. Whitney, Suzanne C. Dufour, Andrew S. Lang

**Affiliations:** 1 Department of Biology, Memorial University of Newfoundland, 232 Elizabeth Ave., St. John’s, NL A1B 3X9, Canada; verhoevenjtp@googlemail.com (J.T.P.V.); munro.hannah@gmail.com (H.J.M.); sheenaroul@hotmail.com (S.R.); hughwhitneynl@gmail.com (H.G.W.); sdufour@mun.ca (S.C.D.); 2 Animal Health Laboratory, Laboratory Services Division, University of Guelph, 419 Gordon St., Guelph, ON N1G 2W1, Canada; dojkic@uoguelph.ca; 3 Wildlife Research Division, Environment and Climate Change Canada, 6 Bruce Street, Mount Pearl, NL A1N 4T3, Canada; greg.robertson@canada.ca

**Keywords:** parvovirus, chaphamaparvovirus, densovirus, avian viruses, insect viruses, virus discovery, codon usage, dinucleotide frequencies

## Abstract

Parvoviruses are small single-stranded DNA viruses that can infect both vertebrates and invertebrates. We report here the full characterization of novel viruses we identified in ducks, including two viral species within the subfamily *Hamaparvovirinae* (duck-associated chapparvovirus, DAC) and a novel species within the subfamily *Densovirinae* (duck-associated ambidensovirus, DAAD). Overall, 5.7% and 21.1% of the 123 screened ducks (American black ducks, mallards, northern pintail) were positive for DAC and DAAD, respectively, and both viruses were more frequently detected in autumn than in winter. Genome organization and predicted transcription profiles of DAC and DAAD were similar to viruses of the genera *Chaphamaparvovirus* and *Protoambidensovirus*, respectively. Their association to these genera was also demonstrated by subfamily-wide phylogenetic and distance analyses of non-structural protein NS1 sequences. While DACs were included in a highly supported clade of avian viruses, no definitive conclusions could be drawn about the host type of DAAD because it was phylogenetically close to viruses found in vertebrates and invertebrates and analyses of codon usage bias and nucleotide frequencies of viruses within the family *Parvoviridae* showed no clear host-based viral segregation. This study highlights the high parvoviral diversity in the avian reservoir with many avian-associated parvoviruses likely yet to be discovered.

## 1. Introduction

Parvoviruses (family *Parvoviridae*, order *Piccovirales*) are small, non-enveloped, icosahedral virions comprised of capsid viral proteins (VPs) surrounding a linear, single-stranded DNA genome with a size range of approximately 4–6 kb. The genomes include two main coding regions with the one in 5′ encoding non-structural (NS) proteins and the one in 3′ coding for the VPs. These are included between two short non-coding palindromic regions that fold into terminal hairpin structures, which are similar in homotelomeric viruses and differ from one another in heterotelomeric viruses. Parvoviruses maximize their genome usage by using alternative splicing to generate multiple mRNAs, which are capped and polyadenylated, that are then translated into the different viral proteins. These include the replication initiator protein NS1, ancillary proteins essential for various stages of the virus life cycle (NS2-4 and NP1), and a variable number of VPs, the smallest of which (VP2-5) share the same sequence with the largest VP1 but present different degrees of N-terminal truncations [1].

Viruses within the family *Parvoviridae* are currently grouped into three phylogenetically defined subfamilies: *Parvovirinae* (10 genera), which includes thus far only viruses infecting vertebrates; *Densovirinae* (11 genera), which comprises viruses infecting invertebrates; and *Hamaparvovirinae*, a recently established taxon that contains viruses identified in both vertebrate (2 genera) and invertebrate (3 genera) hosts [2,3]. This diversity of host species is also matched by a high genetic diversity among viral members, which are characterized by a wide array of genome arrangements, including monosense and ambisense gene organizations, and of auxiliary proteins. Furthermore, their capsid proteins are very divergent and it has been speculated that they might have originated from different ancestral proteins [4], although the unique region of the VP1 from most genera across subfamilies contains a conserved phospholipase A_2_ (PLA_2_) enzymatic domain [1,4,5]. What all parvoviruses have in common, however, is the presence of a highly conserved helicase superfamily 3 (SF3) domain, with helicase and ATPase activity, within the NS1. As a result of its high sequence conservation, this domain is used to define the phylogenetic relationships among all parvoviruses and to establish family-wide taxonomy [2].

Several parvoviruses have been identified in the avian reservoir, but probably the most famous examples are viruses within the species *Anseriform dependoparvovirus 1* (genus *Dependoparvovirus*, subfamily *Parvovirinae*). These include the goose parvovirus (GPV) and the Muscovy duck parvovirus (MDPV) that cause Derzsy’s disease, a fatal disease characterized by watery diarrhea, lethargy, anorexia and prostration in goslings and ducklings (GPV) and Muscovy ducklings (MDPV), and an emerging disease known as short beak and dwarfism syndrome in mule ducks [6]. Within the same genus is the much less characterized and non-pathogenic avian adeno-associated virus (species *Avian dependoparvovirus 1*) [7]. Also within the *Parvovirinae* are the chicken parvovirus and turkey parvovirus (*Galliform aveparvovirus 1*) that have been reported in association with various enteric syndromes, as well as in healthy gut viromes, and are classified within the genus *Aveparvovirus* together with the two recently discovered red-crowned crane parvovirus (*Gruiform aveparvovirus 1*) and pigeon parvovirus (*Columbid aveparvovirus 1*) [3,6,8,9].

In recent years, several avian parvoviruses have been identified within the subfamily *Hamaparvovirinae*. All these viruses are included within the recently established genus *Chaphamaparvovirus*, which takes its name from the host groups in which its members were initially discovered (chiropteran, avian, and porcine) and so far includes viruses identified in mammals and birds [2,3], although recent studies also report phylogenetically related viruses in fish and reptiles [10,11]. Currently this genus includes 16 species, with six identified in the avian reservoir. Specifically, the *Psittacine chaphamaparvovirus 1* that was found through metagenomics analysis in feces of parakeet [12] and the *Galliform chaphamaparvovirus 1-5* that have been found in feces of turkeys and chickens [13,14,15] and deceased peafowls [16]. Furthermore, chaphamaparvoviruses have been reported in fecal specimens collected from red-crowned cranes [9] and ducks [17] and there is in silico evidence for the presence of these viruses in canaries and mesites [18].

The majority of avian parvoviruses have been discovered in recent years thanks to the widespread application of metagenomic sequencing and the number of known species keeps increasing every year [2,3]. It is, therefore, likely that what is currently known is a fraction of the overall viral diversity and a vast repertoire of avian parvoviruses is still waiting to be discovered. This is especially likely considering that there are over 10,000 living avian species [19] and parvoviruses have been investigated in only a few of these. In this study we molecularly characterized novel parvoviruses from two different subfamilies, *Hamaparvovirinae* and *Densovirinae*, that we discovered in a population of wild North American ducks and studied their diversity, distribution, and genomic features.

## 2. Materials and Methods

### 2.1. Sample Collection

This study included archived samples from 144 birds collected for other studies in 2014 (*n* = 92), 2015 (*n* = 39), and 2018 (*n* = 13) in Newfoundland and Labrador, Canada [20,21,22]. Sampling was performed in accordance with the recommendations of the Canadian Council on Animal Care.

The majority of samples were collected at different locations next to ponds within the city of St. John’s (32 at Quidi Vidi Lake, 107 in Bowring Park, 2 at Burton’s Pond, 1 at Commonwealth Pond) and 2 birds came from a nearby city (Bay Roberts). Sampled birds included: 109 American black ducks (*Anas rubripes*), 9 mallards (*Anas platyrhynchos*), 1 northern pintail (*Anas acuta*), 4 American black duck × mallard hybrids, 8 American herring gulls (*Larus smithsonianus*), 9 ring-billed gulls (*Larus delawarensis*), and 4 Iceland gulls (*Larus glaucoides*). Most birds were adults (102/141, 72.3%), and the ducks were evenly distributed between sexes (63/120 female, 52.5%) while sex was not recorded for gulls. Samples were paired oropharyngeal and cloacal swabs (polyester swabs, Starplex Scientific, Etobicoke, ON, Canada) preserved together (samples from 2014 and 2015) or separately (samples from black ducks collected in 2018) into 3 mL universal transport medium (Starswab Multitrans System, Starplex Scientific, Etobicoke, ON, Canada). Additionally, seven serum samples from American black ducks were also included. All animals appeared healthy and showed no signs of disease.

### 2.2. Virus Discovery

The chapparvovirus strain B6 was discovered with the ViDiT (Virus Discovery with Ion Torrent) method and its discovery is described in [23] while the densovirus strain BE8 was discovered with the VidION method, an adaptation of ViDiT for MinION (Oxford Nanopore Technologies, Oxford, UK) sequencing. Briefly, sample pre-treatment (centrifugation and DNase treatment) and nucleic acid (NA) isolation with the DNeasy Blood and Tissue Kit (Qiagen, Hilden, Germany) were performed according to the ViDiT protocol, while cDNA was prepared with the M-MLV Reverse Transcriptase (Promega Madison, WI, USA) and random hexamers using 12 μL NA as input. Library preparation, which included three different polymerase chain reactions (PCRs; random amplification, sequencing library generation, library enrichment) followed by AMPure XP (Beckman Coulter, Brea, CA, USA) purifications (with a ratio of bead solution:sample of 0.7:1 v:v) was performed according to the ViDiT protocol with primers modified to be compatible with MinION sequencing (Supplementary Table S1). Additional modifications to the protocol included an extension time of 1 min instead of 30 s in all PCRs and annealing temperatures of 45 °C and 57 °C during the sequencing library generation PCR and 50 °C during the enrichment PCR. The MinION PCR Barcoding Expansion 1-96 kit (Oxford Nanopore Technologies, Oxford, UK) was used to barcode samples in a PCR mix containing 15 μL library, 15 μL DreamTaq Green PCR Master Mix (ThermoFisher Scientific, Waltham, MA, USA) and 0.6 μL primer. The reaction was performed for 25 cycles using an annealing temperature of 62 °C and an extension time of 1 min. Library concentration was measured with the Qubit™ dsDNA HS Assay Kit (ThermoFisher Scientific, Waltham, MA, USA) and sequencing was performed with the Ligation Sequencing Kit (SQK-LSK109) on a Flow Cell (R9) using a MinION sequencer (Oxford Nanopore Technologies, Oxford, UK). Raw sequence data were base-called and demultiplexed using the Guppy base caller software (Oxford Nanopore Technologies, v4.0.15). Subsequently, National Center for Biotechnology Information (NCBI) DustMasker 1.0.0 [24] was used to detect low complexity regions within sequences which, if found, were trimmed and used as breakpoints to split sequences into multiple high-quality sub-sequences. Resulting sequences were then compared to the NCBI nucleotide database (retrieved 21 June 2020) using the standalone BLASTn 2.11.0+ application from the NCBI BLAST+ suite (package: blast 2.11.0, build October 6, 2020) [25], with settings as described in [23].

### 2.3. Screening and Sequencing

Primers were designed based on fragments identified during virus discovery and used for viral screening through hemi-nested PCRs. In the case of chapparvoviruses, degenerate primers were designed keeping into account sequences of other closely related avian viruses (*Galliform chaphamaparvovirus 2* and *3*) and primers AvChap_F (5′-GGAYTWGGWAAGTGYTGTCC-3′) and AvChap_R1 (5′-GTCCTTYTTGATTHKGACACC-3′) were used during the first PCR to amplify a 259-nt fragment, while primers AvChap_F and AvChap_R2 (GTGTNCKWGG- TAACATATAYGG-3′) were used during the nested PCR to amplify a 202-nt fragment. For the densovirus, primers DensoSc_F1 (5′-CTCTCCCATAGGAACATTTCC-3′) and DensoSc_R (5′-GGAGTACAACCAGTTCCAGC-3′) were used for the first PCR (amplified product size: 174 nt), and primers DensoSc_F2 (5′-GCGTAAGGCCATGCGGTTGG-3′) and DensoSc_R were used for the hemi-nested amplification (amplified product size: 146 nt). Screening PCRs were performed with the DreamTaq Green PCR Master Mix (ThermoFisher Scientific, Waltham, MA, USA) using NA isolated from swab samples available from previous investigations [20,21,22] and from NA isolated from 150 μL sera with the DNeasy Blood and Tissue Kit (Qiagen Hilden, Germany). All positive samples were confirmed by Sanger sequencing.

The complete genome sequences of the identified viruses were obtained through the genome walking approach ViDiWa described in [20]. An attempt to obtain the complete coding sequences of different strains was performed for all chapparvovirus-positive samples and a few densovirus-positive samples through a combination of genome walking and specific PCRs performed with primers designed on already sequenced genomes. Finally, a 722-nt fragment was obtained from several densoviruses to study their molecular epidemiology through hemi-nested PCRs with primer pairs Denso_F14 (5′-TGCAACACGTGTGTTGAGCC-3′) and DensoSc_R, and Denso_F14 and Denso_R1 (5′-AGATACTCGTGCGTATTGGG-3′). All amplified products were purified with AMPure XP beads and outsourced for Sanger sequencing.

### 2.4. Sequence and Phylogenetic Analyses

Sequence analyses and annotations were performed in Geneious R11 (Biomatters, Auckland, New Zealand). Splicing donor and acceptor site prediction was achieved with NNSPLICE 0.9 [26] and promoter prediction with NNPP 2.2 [27]. Similarity plots were performed with Simplot 3.5 [28].

For the phylogenetic analysis of the *Hamaparvovirinae* a dataset was built with all reference sequences of viruses within this subfamily plus all full chaphamaparvoviral NS1 sequences identified in GenBank by a BLASTn search on 21 October 2020 using the settings described in [23]. An exception was the porcine parvovirus 7 for which only two of the 60 highly identical sequences were included. The final dataset contained 95 sequences and their accession numbers are available in Supplementary Figures S1 and S2. For the phylogenetic analysis of the *Densovirinae* we built a dataset with all reference sequences of viruses within this subfamily plus all full NS1 sequences identified through a BLASTp search (expect threshold: 10; word size: 3; gap costs existence: 6, extension:2) on 9 November 2020 that showed homology to the virus of this study with >70% sequence coverage. After removing 17 sequences that were listed in the database as invertebrate DNA/RNA and could be derived from endogenous parvoviral elements, a final set of 114 unique sequences was selected and their accession numbers are available in Supplementary Figure S3.

Protein alignments were performed with MAFFT 7.450 [29] with the L-INS-I algorithm while nucleotide alignments were performed with ClustalW 2.1 [30] and alignments were manually trimmed to remove regions with extended gaps and poorly aligned areas. Maximum-likelihood trees were inferred with IQ-TREE 2 [31] using the best model for genetic distance estimates identified as the one with the lowest BIC (Bayesian information criterion) with the ModelFinder function and both ultrafast bootstrap approximation (ufBoot) [32] and SH-like approximate likelihood ratio test (SH-aLRT) [33] were used to assess branch support. Trees were annotated with FigTree (http://tree.bio.ed.ac.uk/software/figtree/, downloaded on 7 November 2020) and final figures prepared with INKSCAPE (https://inkscape.org/, downloaded on 19 June 2020).

### 2.5. Codon Usage and Nucleotide Frequency Analyses

For these analyses we built a database of 266 sequences, which included the full NS1 and VP1 coding sequences from 133 reference viral species [3] from all the three subfamilies for which the host was known.

Sequences were imported into R (version 4.0.2) for the analyses [34]. The GC content for each sequence was calculated as number of guanine (G) and cytosine (C) over the total number of nucleotides using the GC function available within the seqinR R package version 4.2.4 [35] and data were visualized with box-and-whisker plots using ggplot2 [36]. The relative synonymous codon usage (RSCU) quantifies the influence of a synonymous codon without the confounding influence of amino acid composition and sequence length and is defined as the ratio of the observed frequency of codons to the expected usage frequency under the assumption that all codons for the particular amino acid are used equally [37]. The RSCU for each codon was calculated for all sequences using the seqinR uco function, and relationships of codon usage patterns among different viruses were determined through principal component analyses (PCA), using the prcomp function, to establish if viruses of vertebrates and those of invertebrates have differences in codon usage bias, which would allow the use of this property to predict the parvovirus host type [38]. The recently proposed synonymous dinucleotide usage (SDU), which calculates the ratios of the observed proportion of synonymous dinucleotides to that expected under equal synonymous codon usage for a given dinucleotide frame position, was used in a similar way [39]. The dinuq python package version 1.1.1 [39] was used to calculate the SDU values for each sequence, which were then imported into R and used for PCA analyses. Final figures were edited with INKSCAPE.

### 2.6. Statistical Analyses

Differences among viral positivity rates in different groups (number of positive animals over the total number of individuals) were performed using R 4.0.2 [34]. Data were analyzed using a generalized linear model with a binomial distribution, a logit link function, and categorial response variables. Variables considered were sampling location, year, season, sex, and age. Positivity rates were only analyzed for ducks, as rates were not high enough to make meaningful comparisons for gulls. Due to the unbalanced distribution of data in some categories (e.g., not all locations were sampled in every year and season) analyses were performed on relevant subsets of the data that were reasonably well balanced.

## 3. Results

During a previous virus discovery study performed with the in-house developed method ViDiT on oropharyngeal–cloacal swabs collected from 36 wild birds, including 8 ducks, a 221-nt fragment was identified in one sample that showed homology to viruses within the recently established genus *Chaphamaparvovirus* [23]. Later, during the development of the VidION method, during a test run performed on three samples (two samples from ducks and one from a gull) a second parvoviral fragment (416 nt) was discovered that showed homology to viruses within the subfamily *Densovirinae*. We named these viruses duck-associated chapparvovirus (DAC) and duck-associated ambidensovirus (DAAD).

### 3.1. Positivity Rates

After the initial discovery of DAC and DAAD, we screened archived NA isolated from samples collected from 123 ducks. Overall, DAC and DAAD were identified in 5.7% and 21.1% of samples, respectively, and both viruses were detected in both mallards and American black ducks. Additionally, DAAD was also identified in one sample available from a northern pintail (Table 1). One ring-billed gull also tested chaphamaparvovirus-positive, but subsequent sequence analyses revealed that the gull was infected with a different virus (see below). Both DAC and DAAD showed higher positivity rates during the autumn months (September-November) compared to the winter months (February-April), but these differences were not statistically significant (Supplementary Table S2).

Positivity rates for DAC varied significantly between locations and 27.3% (3/11) of ducks sampled at Quidi Vidi Lake were positive, while only 1.9% (2/107) of the ducks sampled at Bowring Park tested positive (χ^2^ = 9.16, df = 1, *p* = 0.002). Furthermore, the two samples from Bay Roberts were also positive. Six of the seven positive samples were collected in 2015 but samples from 2014 and 2018 were mostly collected from low-positivity areas. Finally, 4 of the 7 positives were adults, 4 were females, 2 were males, and the sex of one was unknown. Differences in positivity rates between males and females and adults and juveniles were not statistically significant (Supplementary Table S2). DAC was only found in 2014 and 2015, with the positivity rate not differing significantly between years (Supplementary Table S2). Two animals presented co-infection by two DAC strains, and, based on previous results, four animals showed co-infections with a different virus. Specifically, one animal was also infected with influenza A virus [22] and three animals presented co-infection with DAAD and a third virus (influenza A virus, duck calicivirus B6, and duck papillomavirus 3) [20,21,22] (Supplementary Table S3).

The positivity rate for DAAD was also higher at Quidi Vidi Lake (5/11, 45.5%) compared to Bowring Park (21/107, 19.6%), but the difference was not significant, and the virus was not found in any other location. DAAD was detected in all sampled years, and the positivity rate differed significantly between years (χ^2^ = 6.91, df = 2, *p* = 0.02). There were no differences in positivity rates considering sex or age of the ducks (Supplementary Table S2). Six of the infected animals were juveniles, 19 were adults, 9 were females, and 15 were males. Six animals presented double infections, two with influenza A virus [22] and four with duck papillomavirus-3 [20], and three animals presented triple infections (Supplementary Table S3). For three positive animals the oral and the cloacal swabs were preserved in separate collection tubes and in all cases the cloacal swab was positive, while the oral swab was positive in only two of the three. Finally, serum samples were available for seven positive animals, but none of these tested positive.

### 3.2. Molecular Features of the Novel Chaphamaparvoviruses

#### 3.2.1. Genome Organization

**Figure 1:**
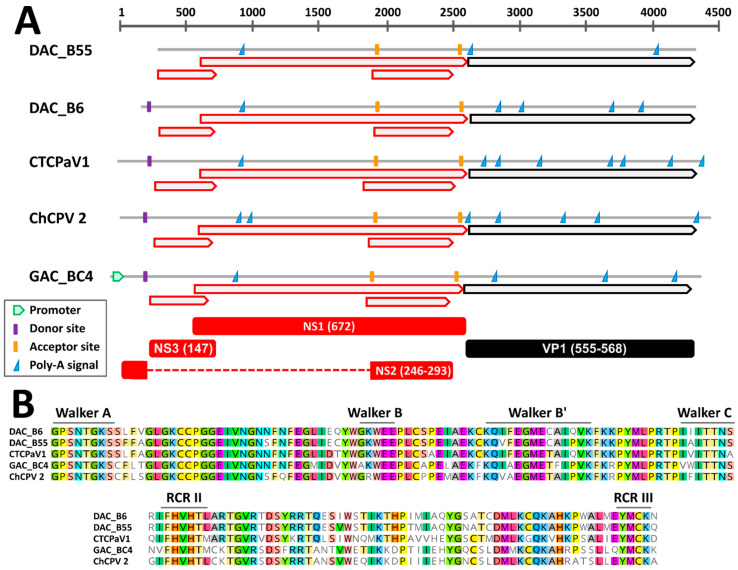
Genomic features of the novel duck-associated chapparvoviruses (DAC) compared to closely related viruses. (**A**) Schematic genome representations with in silico predicted open reading frames (ORFs, red for non-structural proteins (NS) and black for viral proteins (VP)), splicing donor and acceptor sites, poly-adenylation signals, and promoter (as indicated by the legend). Hypothetical proteins (with respective protein sizes) generated after mRNA splicing are shown at the bottom. (**B**) Conserved rolling circle replication (RCR) and Walker motifs typical of parvoviral SF3 helicases. CTCPaV1: Chestnut teal chaphamaparvovirus 1, ChCPV 2: chicken chapparvovirus 2, GAC: gull-associated chapparvovirus.

Through genome walking [20] we managed to obtain the near complete genomic sequence of six viruses, including five DAC strains and a virus identified in a gull. These sequences encompassed the full NS1 and VP1 coding regions while lacking the terminal non-coding portions. By comparing the genomes of viruses closely related to DACs (see below), a similar genome organization could be observed. Specifically, besides the two main open reading frames (ORFs) corresponding to the SF3-domain containing replication initiator protein NS1 and the structural proteins of the capsid, VPs, two additional ORFs overlapping the NS1 ORF were detected. These have been previously reported for chaphamaparvoviruses and they could encode ancillary nonstructural proteins [16,40] (Figure 1A). Finally, the conserved Walker and rolling circle replication (RCR) motifs typical of parvoviral helicases could be distinguished within the deduced NS1 protein sequences (Figure 1B). Chaphamaparvoviruses lack a PLA_2_ domain in VP1.

Based on in silico predictions and by comparing identified signals to expression maps experimentally determined for other avian chaphamaparvoviruses [16], we were able to propose hypotheses about splicing sites and protein expression (Figure 1A). As observed for the peafowl parvovirus 1 (PePV1), we could identify a highly supported (score = 1) donor site at the beginning of the genome, just upstream of the first ORF, and two highly supported acceptor sites, one located at the beginning of the second small ORF (score = 0.85–0.95) and one just upstream of the VP ORF (score = 0.76–0.96). Unfortunately, the genomic sequences of most viruses were incomplete and a potential 5′ promoter could only be identified in the gull virus, while the predicted location of the splicing donor for viruses BE8A, B55, and B57 was outside of the sequenced region. However, this suggests that one unique promoter drives the transcription of all the mRNAs. Finally, the presence of a conserved poly-adenylation signal 197 nt downstream of the stop codon of the first small ORF might indicate the presence of an additional transcript and gives support for the expression of an ancillary protein encoded by this ORF.

#### 3.2.2. Phylogenetic Analyses

**Figure 2:**
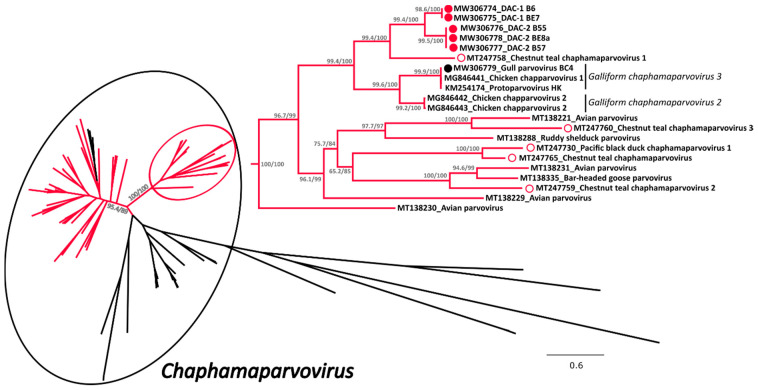
Phylogenetic analysis of the duck associated chapparvoviruses (DAC) within the *Hamaparvovirinae*. The phylogenetic tree based on 101 full NS1 protein sequences was built with the maximum-likelihood method based on the General matrix (LG) + F + R6 model with IQ-Tree [31]. The outcomes of the SH-aLRT and bootstrap test are shown for the main nodes. The branches of the unrooted tree are color-coded based on the host in which viruses have been identified and red represents avian hosts, while black includes all other vertebrate and invertebrate hosts. The black circle indicates viruses within the genus *Chaphamaparvovirus* while the red circle shows the clade containing the viruses studied here, which is also shown enlarged on the right side. The viruses identified in this study are labelled with a colored full circle (red for those found in ducks and black for the one found in a gull), while viruses found by others in ducks (genus *Anas*) are indicated by an empty red circle. Species designations, when available, are indicated on the right.

A phylogenetic tree was built with an alignment of 101 NS1 amino acid sequences of viruses from the subfamily *Hamaparvovirinae* and in this tree the clade defining the genus *Chaphamaparvovirus* was highly supported (Figure 2 and Supplementary Figure S1 for the tree *in extenso*). Within this genus we could observe the presence of two highly supported and closely related clades almost exclusively composed of avian viruses (indicated in red in Figure 2), one of which also contained all the sequences of this study. These included in total 61 sequences from avian viruses and a sub-clade of three sequences identified in Tasmanian devils [41], while no other virus of avian origin was identified in other clades.

All five DACs for which we could obtain the full sequence (indicated by full red circles) clustered together but formed two independent sub-clades (DAC-1 and DAC-2) close to viruses from the species *Galliform chaphamaparvovirus 2* and *3*. Interestingly, viruses recently identified in Australian wild ducks [17] (indicated by an empty red circle) clustered in the same major avian-related clade, but only one of them formed a supported cluster with DACs. In contrast, the virus we identified in a gull was clearly part of the species *Galliform chaphamaparvovirus 3*. Unfortunately, due to co-infections and low viral load, we were not able to obtain the complete sequences of four additional strains, but preliminary analyses based on partial nucleotide sequences show that these viruses are also included in the same avian clade. However, three of them were more divergent and likely more closely related to one of the Australian viruses (Supplementary Figure S2).

Based on the complete NS1 amino acid sequences, DACs were most closely related to the Chestnut teal chaphamaparvovirus 1 (64.5–65.3% identity). Furthermore, the two DAC-1 strains B6 and BE7 presented only one nucleotide difference across the whole genome that resulted in an amino acid substitution in NS1, while DAC-2 strains B55, B57, and BE8a were 98.3–99.2% identical over the whole genome and 98.2–98.5% identical within the NS1 protein. Finally, the identity between DAC-1 and DAC-2 NS1 proteins was 82.5–82.8% and the VP protein was more variable (within-group identity: 96.6–99.3% for DAC-2 and 100% for DAC-1; between-groups identity: 77.8–79.9%).

### 3.3. Molecular Features of the Novel Ambidensoviruses

#### 3.3.1. Genome Organization

The near complete genomic sequence of three DAADs were obtained and these were from American black ducks sampled at two different locations. According to in silico predictions, the genome organization of DAAD was similar to that of the *Culex pipiens* densovirus (CpDNV, species *Dipteran protoambidensovirus 1*), which was experimentally determined [42]. DAAD, in fact, possesses an ambisense gene organization with two predicted promoters, one regulating the expression of the non-structural genes and likely one promoter on the other strand at the other end of the genome regulating the expression of the capsid protein genes. However, the promoter for the expression of capsid protein genes, which was previously determined for CpDNV, could not be identified with the prediction software for any of the viruses.

**Figure 3:**
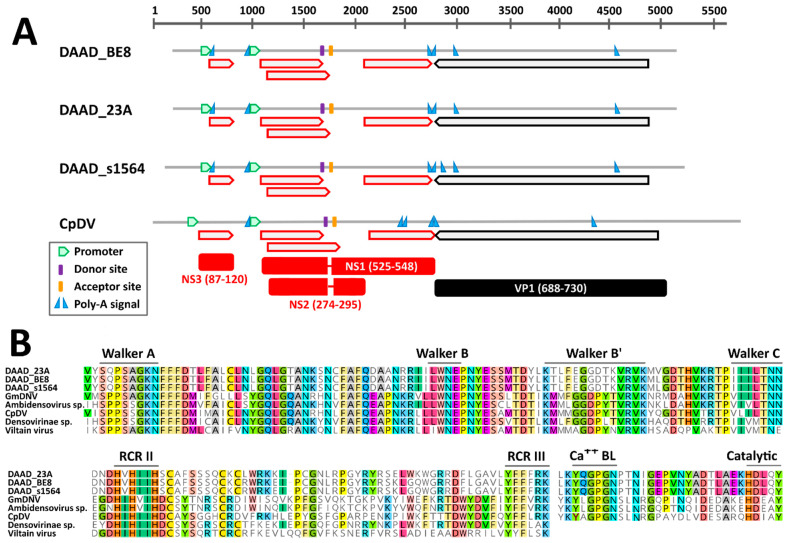
Genomic features of the novel duck-associated ambidensoviruses (DAAD) compared to that of *Culex pipiens* densovirus (CpDV). (**A**) Schematic genome representations with in silico predicted ORFs (red for NS and black for VP proteins), splicing donor and acceptor sites, poly-adenylation signals, and promoter (as indicated by the legend). Hypothetical proteins (with respective protein sizes) generated after mRNA splicing are shown at the bottom. (**B**) Conserved rolling circle replication (RCR) and Walker motifs typical of parvoviral SF3 helicases and phospholipase A_2_ (Ca^++^ BL: calcium binding loop; Catalytic: catalytic site) motifs typical of parvoviral VP1. GmDNV: *Galleria mellonella* densovirus.

While we could identify an intact ORF coding for the capsid proteins, there was no clear full NS1 ORF, but the presence of at least 4 conserved smaller ORFs was observed instead (Figure 3A). However, highly supported splicing donor and acceptor sites, whose positions were conserved in all genomes and experimentally confirmed in CpDNV, were identified and would lead to the creation of two additional ORFs coding for NS1 and NS2. Finally, an additional small ORF downstream of the first promoter could be identified that encodes a hypothetical NS3, although the predicted NS3 proteins of DAAD and CpDNV do not share detectable sequence homology. Finally, although both NS1 and NS2 of DAAD and CpDNV were very dissimilar, we were able to identify in their NS1 proteins the typical motifs of parvoviruses. Specifically, the rolling circle replication (RCR) motifs II and III could be identified at the N-terminal side of the protein and encoded upstream the splicing donor site, while the Walker domains A, B, B’, and C could be identified on the C-terminal side of the protein, encoded downstream of the splicing acceptor site (Figure 3B). This provides support for the predicted genome configuration and splicing pattern. Finally, a PLA_2_ domain was present in the VP1 of DAAD (Figure 3B).

#### 3.3.2. Phylogenetic Analysis

**Figure 4:**
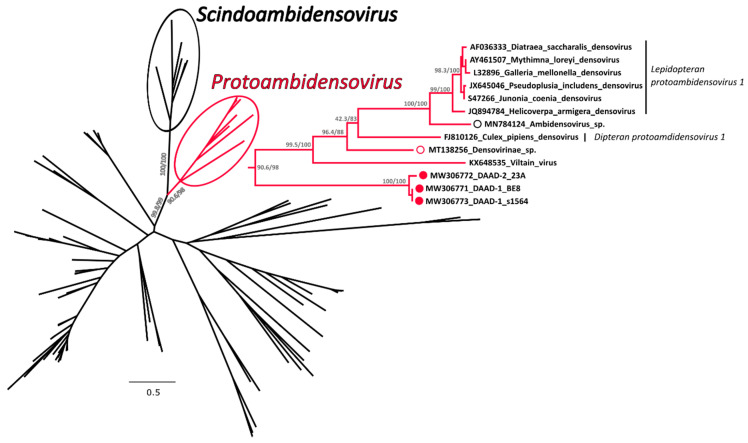
Phylogenetic analysis of the duck associated ambidensovirus (DAAD) within the *Densovirinae*. The phylogenetic tree based on 117 full NS1 protein sequences was built with the maximum-likelihood method based on the General matrix (LG) + F + R6 model with IQ-Tree [31]. The outcomes of the SH-aLRT and bootstrap test are shown for main nodes. The branches of the unrooted tree are color-coded based on the taxonomy and red represents putative members of the genus *Protoambidensovirus* (enclosed in a red circle and shown *in extenso* on the right), while black includes all other viruses. The black circle indicates viruses within the genus *Scindoambidensovirus*. The viruses identified in this study are labelled with a full red circle, while viruses found by others in vertebrates are indicated by an empty red and black circle for avian and mammal studies, respectively. Species designations, when available, are indicated on the right.

A phylogenetic tree was built with an alignment of 117 NS1 amino acid sequences from viruses in the subfamily *Densovirinae* and in this tree DAADs were included in a highly supported clade that also included members of the genus *Protoambidensovirus* (Figure 4 and Supplementary Figure S3 for the tree *in extenso*). This formed another highly supported clade with members of the genus *Scindoambidensovirus*, its closest related group of viruses. Within the protoambidensoviruses, sequences found during metagenomic analyses of samples from birds (indicated by an empty red circle) and a drill monkey living in a wildlife sanctuary in Nigeria [43] (empty black circle) were also included and DAADs (full red circles) were the closest to the root of the clade.

The complete NS1 amino acid sequences of DAADs were equally distant from most members of the *Protoambidensovirus* clade (33.3–34.5% identity) and they were the least identical to the *Drosophila melanogaster* Viltain virus. However, based on a BLASTn analysis, DAADs were the closest to viruses identified via metagenomic investigations of insect-eating Chinese bats [44] (accession numbers JN857346 and JN857337), but these viruses were only partially sequenced and the full NS1 sequences were not available for the phylogenetic analysis.

Among our sequences, we identified two different viral strains, one represented by the sequence 23A and one represented by the two other sequences BE8 and s1564. These two strains were approximately 98% identical to each other but, while the ends of the genome were almost identical, the middle part (between nucleotides 1289 and 2741 of the s1564 strain) was more divergent, a feature that suggests recombination as indicated by the similarity plot (Supplementary Figure S4). Finally, to evaluate the distribution of the two variants, we sequenced an approximately 700 nt-long fragment of the variable region from an additional 10 viruses. We observed that the variant BE8/s1564 was the predominant one as only three animals were positive for the variant 23A, including the northern pintail and an American black duck that presented both variants simultaneously. Both variants were identified at the two different sampling sites.

### 3.4. Codon Usage and Nucleotide Frequency Bias Analyses

**Figure 5:**
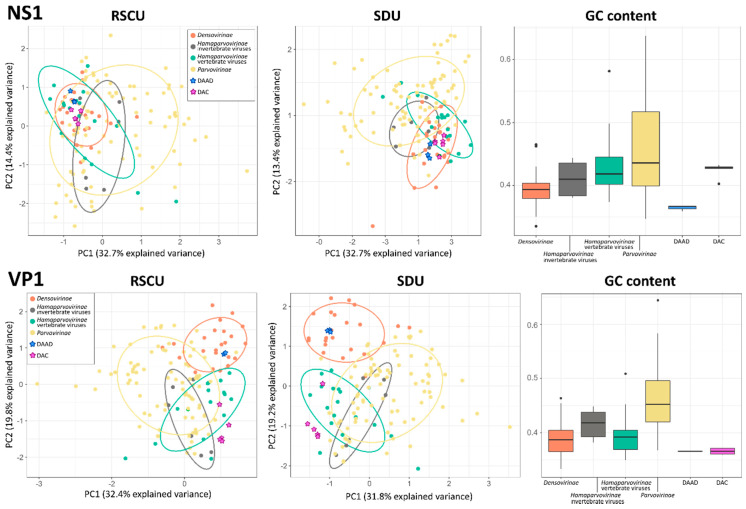
Compositional analyses of viruses within the *Parvoviridae*. Principal component analysis (PCA) plots based on relative synonymous codon usage (RSCU) and synonymous dinucleotide usage (SDU) values are shown on the left and in the center, respectively; ellipses are drawn around groups as indicated in the legend with a normal probability size of 70% and axes represent dimensions explaining the largest variances. Box-and-whisker plots based on GC contents in each group are showed on the right; upper and lower whiskers indicate the highest and lowest values within 1.5 times the interquartile range, while boxes indicate the interquartile range itself with average values displayed as thick lines. Dots indicate potential outliers.

The GC content and the variance among the RSCU and SDU of sequences from 141 parvoviruses, including the eight viruses we describe in this study, were evaluated to determine whether viruses infecting vertebrates and those infecting invertebrates showed different properties and whether we could use this to predict the host of the viruses we discovered. All parameters were calculated for VP1 and NS1 separately and, to account for potential variations both between sub-families and host types, viruses within the *Hamaparvovirinae* were divided into two groups, those infecting vertebrates and those infecting invertebrates (Figure 5).

All analyses performed with VP1 sequences produced clearer separations between groups. In fact, the five groups considered overlapped significantly in the two PCA plots obtained with the NS1 sequence, while a clear distinction between densoviruses and all other groups could be observed in the VP1-based plots with a significant overlap between parvoviruses and hamaparvoviruses, regardless of the host type. In terms of GC content, members of the *Hamaparvovirinae* occupied an intermediate position between parvoviruses and densoviruses, which showed the highest and lowest GC contents, respectively. Interestingly, while the NS1 GC contents for the two hamaparvovirus groups were similar, the VP1 GC contents were lower in vertebrate hamaparvoviruses than in invertebrate hamaparvoviruses, making vertebrate hamaparvoviruses more similar to densoviruses in this respect.

## 4. Discussion

Like many other viral families, the number of known *Parvoviridae* members has experienced substantial growth in recent years with the definition of several novel viral species and genera [2] as more and more novel viral genomes have been discovered, especially thanks to the increasing accessibility of next-generation sequencing techniques [23]. In particular, the discovery of parvoviruses of vertebrates that showed a high genetic identity to viruses of invertebrates, now known as chaphamaparvoviruses, stimulated the very recent definition of a new viral subfamily, the *Hamaparvovirinae*, the only one of the three parvoviral subfamilies to include viruses capable of infecting both vertebrates and invertebrates [2]. The discovery of this lineage not only led to a reevaluation of the ecology and evolutionary history of these parvoviruses [40], but also facilitated the discovery of many viruses that were previously too divergent from known ones to be identifiable on the basis of sequence identity. This is especially true for avian parvoviruses whose number grew significantly in the past few years.

### 4.1. Duck-Associated Chapparvovirus (DAC) and Duck-Associated Ambidensovirus (DAAD) Are Novel Viral Species

In our study we report the molecular characterization of avian parvoviruses belonging to two different viral subfamilies that we discovered within the same duck populations using metagenomic methods [23]. All viruses defined in this study possessed all molecular markers typical of parvoviruses, which include the presence of two main ORFs coding for structural and non-structural proteins and the presence of RCR and helicase domains in the NS1.

One of these viruses, which we named DAAD, belongs to the *Densovirinae* and was identified through a MinION sequencing-based virus discovery approach (VidION). DAAD is highly divergent from all known parvoviruses, sharing only 34.5% NS1 protein sequence identity with its closet relative, the *Mythimna loreyi* densovirus, a member of the genus *Protoambidensovirus*. According to the ICTV demarcation criteria for genera definition [2], DAAD could be considered a member of the genus *Protoambidensovirus* as it is included in a highly supported clade with other members of this genus based on the phylogeny of NS1 amino acid sequences. It also possesses the same genome organization as the CpDV, the best characterized virus in this genus whose protein expression and splicing profile were determined in vitro [42]. All three DAAD genomes showed high genetic identity and could be considered the same novel viral species.

The other viruses, which we identified with the ViDiT method during a previous study [23] and named DAC, belong to the subfamily *Hamaparvovirinae*. They demonstrate higher NS1 identities to their closest relatives (approximately 65%) and were, therefore, undoubtedly considered part of the genus *Chaphamaparvovirus*. The criterion for species definition established by the ICTV defines a species-level NS1 protein identity cut-off of 85% [2] and therefore the chaphamaparvoviruses identified in this study for which a complete genomic sequence was obtained (DAC-1 and DAC-2) have to be considered two separate and novel species. Also, the DAC genome organization is similar to other chaphamaparvoviruses for which the transcription profile has been determined in vitro [16].

### 4.2. Potential Hosts and Epidemiology of DAC and DAAD

Approximately 64% (61/95) of full NS1 chaphamaparvoviral sequences we retrieved from GenBank were labelled as being of avian origin, although most of these sequences originate from metagenomic investigations and are not associated with a published manuscript yet. This huge diversity of avian chaphamaparvoviruses indicates that these viruses are likely pervasive, more diverse than currently recognized, and common in the avian reservoir. All avian chaphamaparvoviruses clustered within two highly supported and closely related clades, indicating a strong virus-host relationship. This was also revealed by the presence of smaller clades that included viruses infecting closely related birds, as seen for the viruses of turkeys and peafowls and the close relationships among viruses from ducks. Interestingly, DAC-1 and DAC-2 were closely related to a virus identified in chestnut teals in Australia [17], indicating that genetically related viruses circulate among closely related birds that live long distances apart and that, potentially, bird migration has a role in virus dispersal.

There were many other instances, however, of closely related viruses identified in birds from different orders (e.g., Passeriformes and Psittaciformes). One example is the *Galliform chaphamaparvovirus 3*, a virus originally identified in chickens (order Galliformes) that we also found in a gull (order Charadriiformes). The positive gull was sampled at a location which is near a chicken processing plant and this could indicate waste products derived from farmed chicken processing as a possible source of infection. This viral promiscuity is likely linked to the capability of these viruses to infect different bird species when the opportunity for cross-species infections occurs, for example when different birds share the same ecological niche. These epidemiological characteristics of high viral prevalence, frequent co-infection, and relaxed host-specificity are characteristics shared with other avian viruses from other families [21,45,46].

Although the number of tested birds was small and future studies will have to investigate this further, we observed that DAADs and DACs had very similar epidemiological profiles: both were found in ducks but not in gulls, both were identified between September and April, positivity rates for both were higher during autumn compared to winter, both were more frequent at one of the two locations, and no significant differences were identified when considering sex and age of positive ducks for both, although the positivity rate for DAC was a little higher in juvenile birds, which is consistent with previous reports [17]. Furthermore, although positivity rates were high, not all animals were positive, all infected animals showed no sign of disease, and we observed genetic variation among identified strains with no specific segregation of strains between different locations. To clarify whether these viruses are duck pathogens and to establish viral tropism and replication dynamics, follow-up epidemiological investigations in animals showing signs of disease as well as studies evaluating viral presence and distribution in tissues in combination with assessing viral loads in bodily fluids will be required.

Nonetheless, based solely on epidemiological data, it is tempting to label both DAC and DAAD as avian viruses. However, while the phylogenetic placement of the DAC sequences within a clade that is dominated by avian viruses within a genus that includes many vertebrate viruses, some of which even proven to be pathogenic [47,48], clearly points towards ducks as DAC hosts, such a clear conclusion cannot be made for DAAD. Densoviruses are frequently identified during metagenomic investigations of samples collected from vertebrates [43,44,49], including human plasma and cerebrospinal fluid [50,51], and some of the densoviruses genetically close to DAAD were also vertebrate-associated as they were detected in fecal specimens of birds, monkeys, and bats [43,44]. However, dipteran and lepidopteran viruses were also included in the same clade as DAAD and no proof exists that densoviruses can replicate in vertebrate hosts. Furthermore, we found DAAD in both cloacal and oral cavities of ducks, but the negativity of the sera excludes a detectable systemic infection. Unfortunately, the RSCU, SDU, and GC content analyses did not help in determining the host type since no pattern was observed that could discriminate between vertebrate and invertebrate viruses, corroborating previous findings for the *Parvovirinae* [52]. However, results from these analyses partially reflect the phylogenetic relationships of these viral subfamilies and discriminate densoviruses from the other taxa (based on RSCU and SDU analyses performed with VP1) and parvoviruses from the other taxa (based on a higher GC content) and leave hamaparvoviruses as an intermediate group (with a low GC content and a codon usage bias similar to that of parvoviruses). Overall, VP1 performed better at defining groups and this could reflect the alleged paraphyletic origin of this genomic region in these viruses [4].

Although it seems most likely that DAAD is a virus of invertebrates that made its way in samples from ducks through food or parasites, it should not be definitively excluded without further studies that densoviruses could be capable of infecting both vertebrates and invertebrates, once again challenging the paradigm of classical parvovirus segregation based on host association.

## 5. Conclusions

In this study we report the discovery and full molecular characterization of three novel duck-associated parvoviral species belonging to two different subfamilies, expanding our knowledge of parvoviral diversity and distribution. Epidemiological data showed similarities between the distribution of these viruses with infection rates varying among locations and between seasons and with the circulation of multiple strains. However, epidemiology alone was not sufficient to provide conclusive answers about viral hosts, highlighting the importance of examining novel viruses from multiple points of view to be able to draw meaningful conclusions and avoid false predictions [53,54,55]. Molecular characterization and phylogenetic analyses provided detailed information about similarities with other viruses allowing speculations about viral hosts and showed how parvoviral diversity in the avian reservoir is much higher than anticipated with many avian-associated parvoviruses likely yet to be discovered, as also predicted for viruses within other families [20,21].

## Figures and Tables

**Table 1:** Percentage of birds from different species that tested positive for duck-associated chapparvovirus (DAC) and duck-associated ambidensovirus (DAAD).

Host		DAC ^1^	DAAD ^1^
Ducks	Total, *n* = 123	7 (5.7%)	26 (21.1%)
	American black duck, *n* = 109	6 (27.3%)	22 (20.2%)
	Mallard, *n* = 9	1 (11.1%)	2 (22.2%)
	Northern pintail, *n* = 1	0 (0%)	1 (100%)
	Hybrids, *n* = 4	0 (0%)	1 (25%)
Gulls	Total, *n* = 21	0 (0%)	0 (0%)
	Herring gull, *n* = 8	0 (0%)	0 (0%)
	Iceland gull, *n* = 4	0 (0%)	0 (0%)
	Ring-billed gull, *n* = 9	0 (0%)	0 (0%)

^1^ DAC: duck-associated chapparvovirus; DAAD: duck-associated ambidensovirus.

## Data Availability

All sequences obtained in this study have been deposited in GenBank under accession numbers MW306771-MW306779 for complete genomes and MW306780-MW306794 for partial sequences.

## Supplementary Materials

The following are available online at https://www.mdpi.com/1999-4915/13/2/193/s1, Supplementary Table S1: Primers used for VidION, Supplementary Table S2: Duck-associated chapparvovirus (DAC) and duck-associated ambidensovirus (DAAD) positivity rates in different duck groups, Supplementary Table S3: Viruses identified in single and multiple infections, Supplementary Figure S1: Phylogenetic analysis of the duck-associated chapparvovirus (DAC) within the *Hamaparvovirinae*, Supplementary Figure S2: Phylogenetic analyses of the partially sequenced duck-associated chapparvoviruses (DAC), Supplementary Figure S3: Phylogenetic analysis of the duck-associated ambidensovirus (DAAD) within the *Densovirinae*, Supplementary Figure S4: Similarity plot between duck-associated ambidensoviruses (DAAD) identified in this study.

## References

[1] Cotmore S.F., Agbandje-McKenna M., Canuti M., Chiorini J.A., Eis-Hubinger A.-M., Hughes J., Mietzsch M., Modha S., Ogliastro M., Pénzes J.J. (2019). ICTV virus taxonomy profile: *Parvoviridae*. J. Gen. Virol..

[2] Pénzes J.J., Söderlund-Venermo M., Canuti M., Eis-Hübinger A.M., Hughes J., Cotmore S.F., Harrach B. (2020). Reorganizing the family *Parvoviridae*: A revised taxonomy independent of the canonical approach based on host association. Arch. Virol..

[3] Pénzes J.J., Canuti M., Söderlund-Venermo M., Eis-Huebinger A.M., Ogliastro M., Harrach B. (2020). Create three new genera and 19 new species (*Piccovirales*: *Parvoviridae*). ICTV Taxonomy Proposal 2020.

[4] Mietzsch M., Pénzes J.J., Agbandje-McKenna M. (2019). Twenty-five years of structural parvovirology. Viruses.

[5] Cotmore S.F., Tattersall P. (2014). Parvoviruses: Small does not mean simple. Ann. Rev. Virol..

[6] Kapgate S.S., Kumanan K., Vijayarani K., Barbuddhe S.B. (2018). Avian parvovirus: Classification, phylogeny, pathogenesis and diagnosis. Avian Pathol..

[7] Bossis I., Chiorini J.A. (2003). Cloning of an Avian adeno-associated virus (AAAV) and generation of recombinant AAAV particles. J. Virol..

[8] Phan T.G., Vo N.P., Boros Á., Pankovics P., Reuter G., Li O.T.W., Wang C., Deng X., Poon L.L.M., Delwart E. (2013). The viruses of wild pigeon droppings. PLoS ONE.

[9] Wang Y., Yang S., Liu D., Zhou C., Li W., Lin Y., Wang X., Shen Q., Wang H., Li C. (2019). The fecal virome of red-crowned cranes. Arch. Virol..

[10] Chang W.-S., Li C.-X., Hall J., Eden J.-S., Hyndman T.H., Holmes E.C., Rose K. (2020). Meta-transcriptomic discovery of a divergent circovirus and a chaphamaparvovirus in captive reptiles with proliferative respiratory syndrome. Viruses.

[11] Du J., Wang W., Chan J.F.-W., Wang G., Huang Y., Yi Y., Zhu Z., Peng R., Hu X., Wu Y. (2019). Identification of a novel ichthyic parvovirus in marine species in Hainan Island, China. Front. Microbiol..

[12] Duarte M.A., Silva J.M.F., Brito C.R., Teixeira D.S., Melo F.L., Ribeiro B.M., Nagata T., Campos F.S. (2019). Faecal virome analysis of wild animals from Brazil. Viruses.

[13] Reuter G., Boros Á., Delwart E., Pankovics P. (2014). Novel circular single-stranded DNA virus from turkey faeces. Arch. Virol..

[14] Lima D.A., Cibulski S.P., Tochetto C., Varela A.P.M., Finkler F., Teixeira T.F., Loiko M.R., Cerva C., Junqueira D.M., Mayer F.Q. (2019). The intestinal virome of malabsorption syndrome-affected and unaffected broilers through shotgun metagenomics. Virus Res..

[15] Kim H.-R., Kwon Y.-K., Jang I., Bae Y.-C. (2020). Viral metagenomic analysis of chickens with runting-stunting syndrome in the Republic of Korea. Virol. J..

[16] Liu X., Wang H., Liu X., Li Y., Chen J., Zhang J., Wang X., Shen S., Wang H., Deng F. (2020). Genomic and transcriptional analyses of novel parvoviruses identified from dead peafowl. Virology.

[17] Vibin J., Chamings A., Klaassen M., Bhatta T.R., Alexandersen S. (2020). Metagenomic characterisation of avian parvoviruses and picornaviruses from Australian wild ducks. Sci. Rep..

[18] de Souza W.M., Romeiro M.F., Fumagalli M.J., Modha S., de Araujo J., Queiroz L.H., Durigon E.L., Figueiredo L.T.M., Murcia P.R., Gifford R.J. (2017). Chapparvoviruses occur in at least three vertebrate classes and have a broad biogeographic distribution. J. Gen. Virol..

[19] Prum R.O., Berv J.S., Dornburg A., Field D.J., Townsend J.P., Lemmon E.M., Lemmon A.R. (2015). A comprehensive phylogeny of birds (Aves) using targeted next-generation DNA sequencing. Nature.

[20] Canuti M., Munro H.J., Robertson G.J., Kroyer A.N.K., Roul S., Ojkic D., Whitney H.G., Lang A.S. (2019). New insight into avian papillomavirus ecology and evolution from characterization of novel wild bird papillomaviruses. Front. Microbiol..

[21] Canuti M., Kroyer A.N.K., Ojkic D., Whitney H.G., Robertson G.J., Lang A.S. (2019). Discovery and characterization of novel RNA viruses in aquatic North American wild birds. Viruses.

[22] Roul S., Robertson G., Lang A.S. Assessing the Impact of Low-Pathogenicity Avian Influenza Virus on the Health of American Black Ducks (*Anas rubripes*).

[23] Verhoeven J.T.P., Canuti M., Munro H.J., Dufour S.C., Lang A.S. (2018). ViDiT-CACTUS: An inexpensive and versatile library preparation and sequence analysis method for virus discovery and other microbiology applications. Can. J. Microbiol..

[24] Morgulis A., Gertz E.M., Schäffer A.A., Agarwala R. (2006). A fast and symmetric DUST implementation to mask low-complexity DNA sequences. J. Comput. Biol..

[25] Camacho C., Coulouris G., Avagyan V., Ma N., Papadopoulos J., Bealer K., Madden T.L. (2009). BLAST+: Architecture and applications. BMC Bioinform..

[26] Reese M.G., Eeckman F.H., Kulp D., Haussler D. (1997). Improved splice site detection in Genie. J. Comput. Biol..

[27] Reese M.G. (2001). Application of a time-delay neural network to promoter annotation in the *Drosophila melanogaster* genome. Comput. Chem..

[28] Lole K.S., Bollinger R.C., Paranjape R.S., Gadkari D., Kulkarni S.S., Novak N.G., Ingersoll R., Sheppard H.W., Ray S.C. (1999). Full-length human immunodeficiency virus type 1 genomes from subtype C-infected seroconverters in India, with evidence of intersubtype recombination. J. Virol..

[29] Katoh K., Standley D.M. (2013). MAFFT multiple sequence alignment software version 7: Improvements in performance and usability. Mol. Biol. Evol..

[30] Larkin M.A., Blackshields G., Brown N.P., Chenna R., McGettigan P.A., McWilliam H., Valentin F., Wallace I.M., Wilm A., Lopez R. (2007). Clustal W and Clustal X version 2.0. Bioinformatics.

[31] Minh B.Q., Schmidt H.A., Chernomor O., Schrempf D., Woodhams M.D., von Haeseler A., Lanfear R. (2020). IQ-TREE 2: New models and efficient methods for phylogenetic inference in the genomic era. Mol. Biol. Evol..

[32] Hoang D.T., Chernomor O., von Haeseler A., Minh B.Q., Vinh L.S. (2018). UFBoot2: Improving the ultrafast bootstrap approximation. Mol. Biol. Evol..

[33] Guindon S., Dufayard J.-F., Lefort V., Anisimova M., Hordijk W., Gascuel O. (2010). New algorithms and methods to estimate maximum-likelihood phylogenies: Assessing the performance of PhyML 3.0. Syst. Biol..

[34] R Core Team (2020). R: A Language and Environment for Statistical Computing.

[35] Charif D., Lobry J.R., Bastolla U., Porto M., Roman H.E., Vendruscolo M. (2007). SeqinR 1.0-2: A contributed package to the R project for statistical computing devoted to biological sequences retrieval and analysis. Structural Approaches to Sequence Evolution: Molecules, Networks, Populations.

[36] Wickham H. (2016). ggplot2: Elegant Graphics for Data Analysis.

[37] Sharp P.M., Li W.-H. (1986). An evolutionary perspective on synonymous codon usage in unicellular organisms. J. Mol. Evol..

[38] Wong E.H., Smith D.K., Rabadan R., Peiris M., Poon L.L. (2010). Codon usage bias and the evolution of influenza A viruses. Codon Usage Biases of Influenza Virus. BMC Evol. Biol..

[39] Lytras S., Hughes J. (2020). Synonymous dinucleotide usage: A codon-aware metric for quantifying dinucleotide representation in viruses. Viruses.

[40] Pénzes J.J., de Souza W.M., Agbandje-McKenna M., Gifford R.J. (2019). An ancient lineage of highly divergent parvoviruses infects both vertebrate and invertebrate hosts. Viruses.

[41] Chong R., Shi M., Grueber C.E., Holmes E.C., Hogg C.J., Belov K., Barrs V.R. (2019). Fecal viral diversity of captive and wild tasmanian devils characterized using virion-enriched metagenomics and metatranscriptomics. J. Virol..

[42] Baquerizo-Audiot E., Abd-Alla A., Jousset F.-X., Cousserans F., Tijssen P., Bergoin M. (2009). Structure and expression strategy of the genome of *Culex pipiens* densovirus, a mosquito densovirus with an ambisense organization. J. Virol..

[43] George U., Simsek C., Faleye T.O.C., Arowolo O., Oragwa A., Adewumi O.M., Matthijnssens J., Adeniji J.A. (2020). Genome sequences of novel members of previously described DNA and RNA virus families, isolated from feces of a drill monkey in Nigeria. Microbiol. Resour. Announc..

[44] Ge X., Li Y., Yang X., Zhang H., Zhou P., Zhang Y., Shi Z. (2012). Metagenomic analysis of viruses from bat fecal samples reveals many novel viruses in insectivorous bats in China. J. Virol..

[45] Wille M., Holmes E.C. (2020). Wild birds as reservoirs for diverse and abundant gamma- and deltacoronaviruses. FEMS Microbiol. Rev..

[46] Venkatesh D., Poen M.J., Bestebroer T.M., Scheuer R.D., Vuong O., Chkhaidze M., Machablishvili A., Mamuchadze J., Ninua L., Fedorova N.B. (2018). Avian influenza viruses in wild birds: Virus evolution in a multihost ecosystem. J. Virol..

[47] Edmondson E.F., Hsieh W.-T., Kramer J.A., Breed M.W., Roelke-Parker M.E., Stephens-Devalle J., Pate N.M., Bassel L.L., Hollingshead M.G., Karim B.O. (2020). Naturally acquired mouse kidney parvovirus infection produces a persistent interstitial nephritis in immunocompetent laboratory mice. Vet. Pathol..

[48] Lee Q., Padula M.P., Pinello N., Williams S.H., O’Rourke M.B., Fumagalli M.J., Orkin J.D., Song R., Shaban B., Brenner O. (2020). Murine and related chapparvoviruses are nephro-tropic and produce novel accessory proteins in infected kidneys. PLoS Pathog..

[49] Conceição-Neto N., Godinho R., Álvares F., Yinda C.K., Deboutte W., Zeller M., Laenen L., Heylen E., Roque S., Petrucci-Fonseca F. (2017). Viral gut metagenomics of sympatric wild and domestic canids, and monitoring of viruses: Insights from an endangered wolf population. Ecol. Evol..

[50] Phan T.G., Messacar K., Dominguez S.R., da Costa A.C., Deng X., Delwart E. (2016). A new densovirus in cerebrospinal fluid from a case of anti-NMDA-receptor encephalitis. Arch. Virol..

[51] Fahsbender E., da-Costa A.C., Gill D.E., Milagres F.A.d.P., Brustulin R., Monteiro F.J.C., Rego M.O.d.S., Ribeiro E.S.D., Sabino E.C., Delwart E. (2020). Plasma virome of 781 Brazilians with unexplained symptoms of arbovirus infection include a novel parvovirus and densovirus. PLoS ONE.

[52] Sirihongthong T., Jitobaom K., Phakaratsakul S., Boonarkart C., Suptawiwat O., Auewarakul P. (2019). The relationship of codon usage to the replication strategy of parvoviruses. Arch. Virol..

[53] Canuti M., van der Hoek L. (2014). Virus discovery: Are we scientists or genome collectors?. Trends Microbiol..

[54] Canuti M., Deijs M., Jazaeri Farsani S.M., Holwerda M., Jebbink M.F., de Vries M., van Vugt S., Brugman C., Verheij T., Lammens C. (2014). Metagenomic analysis of a sample from a patient with respiratory tract infection reveals the presence of a γ-papillomavirus. Front. Microbiol..

[55] Naccache S.N., Greninger A.L., Lee D., Coffey L.L., Phan T., Rein-Weston A., Aronsohn A., Hackett J., Delwart E.L., Chiu C.Y. (2013). The perils of pathogen discovery: Origin of a novel parvovirus-like hybrid genome traced to nucleic acid extraction spin columns. J. Virol..

