# Supplementary material for: Investigating the Diversity and Host Range of Novel Parvoviruses from North American Ducks Using Epidemiology, Phylogenetics, Genome Structure, and Codon Usage Analysis
Source: Viruses. 2021 Jan 28;13(2):193. doi: 10.3390/v13020193 (PMC7912424; doi:10.3390/v13020193)

Investigating the diversity and host range of novel parvoviruses from North American ducks using epidemiology, phylogenetics, genome structure, and codon usage analysis

Marta Canuti, Joost T. P. Verhoeven, Hannah J. Munro, Sheena Roul, Davor Ojkic, Gregory J. Robertson, Hugh G. Whitney, Suzanne C. Dufour and Andrew S. Lang

Table of content

Supplementary Table S1. Primers used for VidION. 2

Supplementary Table S2. Duck-associated chapparvovirus (DAC) and duck-associated ambidensovirus (DAAD) positivity rates in different duck groups. 2

Supplementary Table S3. Viruses identified in single and multiple infections........ 2

Supplementary Figure S1. Phylogenetic analysis of the duck-associated chapparvovirus (DAC) within the *Hamaparvovirinae*. 3

Supplementary Figure S2. Phylogenetic analyses of the partially sequenced duck-associated chapparvoviruses (DAC). 4

Supplementary Figure S3. Phylogenetic analysis of the duck-associated ambidensovirus (DAAD) within the *Densovirinae*. 5

Supplementary Figure S4. Similarity plot between duck-associated ambidensoviruses (DAAD) identified in this study........ 6

Supplementary Table S1. Primers used for VidION.

Step	Primer name	Primer sequence 5′→3′
Random amplification	VDNF	GTTGGTGCTGATATTGCNNNNNNNN
	VDNR	CCTGTCGCTCTATCTTCNNNNNNNN
Sequencing library generation	VDF	TCATCTCATCTTTCTGTTGGTGCTGATATTGC
	VDR	CCTCTCTATGACTTGCCTGTCGCTCTATCTTC
Library enrichment	MIF	TTTCTGTTGGTGCTGATATTGC
	MIR	ACTTGCCTGTCGCTCTATCTTC

Supplementary Table S2. Duck-associated chapparvovirus (DAC) and duck-associated ambidensovirus (DAAD) positivity rates in different duck groups.

Variable		DAC	DAAD
Age	Adult, N=89	4 (4.5%)	19 (21.4%)
	Juvenile, N=31	3 (9.7%)	6 (19.4%)
Sex	Female, N=63	4 (6.3%)	9 (14.3%)
	Male, N=58	2 (3.5%)	15 (25.9%)
Season	Autumn[1], N=81	6 (7.4%)	20 (24.7%)
	Winter[1], N=41	1 (2.4%)	6 (14.6%)
Location	Quidi Vidi Lake, N=11	3 (27.3)	5 (45.5%)
	Bowring Park, N=107	2 (1.9%)	21 (19.6%)
Year	2014, N=91	1 (1.1%)	14 (15.4%)
	2015, N=18	6 (33.3%)	8 (44.4%)
	2018, N=12	0 (0.0%)	3 (25.0%)

[1] Autumn: September to November, Winter: February to April.

Supplementary Table S3. Viruses identified in single and multiple infections.

		N	%
DAC, N=7	DAC single infection	3	42.9
	DAC + avian influenza virus	1	14.3
	DAC + DAAD + avian influenza virus	1	14.3
	DAC + DAAD + duck calicivirus B6	1	14.3
	DAC + DAAD + duck papillomavirus 3	1	14.3
DAAD, N=26	DAAD single infections	17	65.4
	DAAD + duck papillomavirus 3	4	15.4
	DAAD + avian influenza virus	2	7.7
	DAAC + DAD + avian influenza virus	1	3.8
	DAAC + DAD + duck calicivirus B6	1	3.8
	DAAC + DAD + duck papillomavirus 3	1	3.8

Supplementary Figure S1. Phylogenetic analysis of the duck-associated chapparvovirus (DAC) within the *Hamaparvovirinae*.
The phylogenetic tree based on 101 full NS1 protein sequences was built with the maximum-likelihood method based on the LG+F+R6 model with IQ-Tree. The outcome of the SH-aLRT and bootstrap test are shown for the main nodes. The branches of the tree are color-coded based on the host in which viruses have been identified and red represents avian hosts, while black includes all other vertebrate and invertebrate hosts.

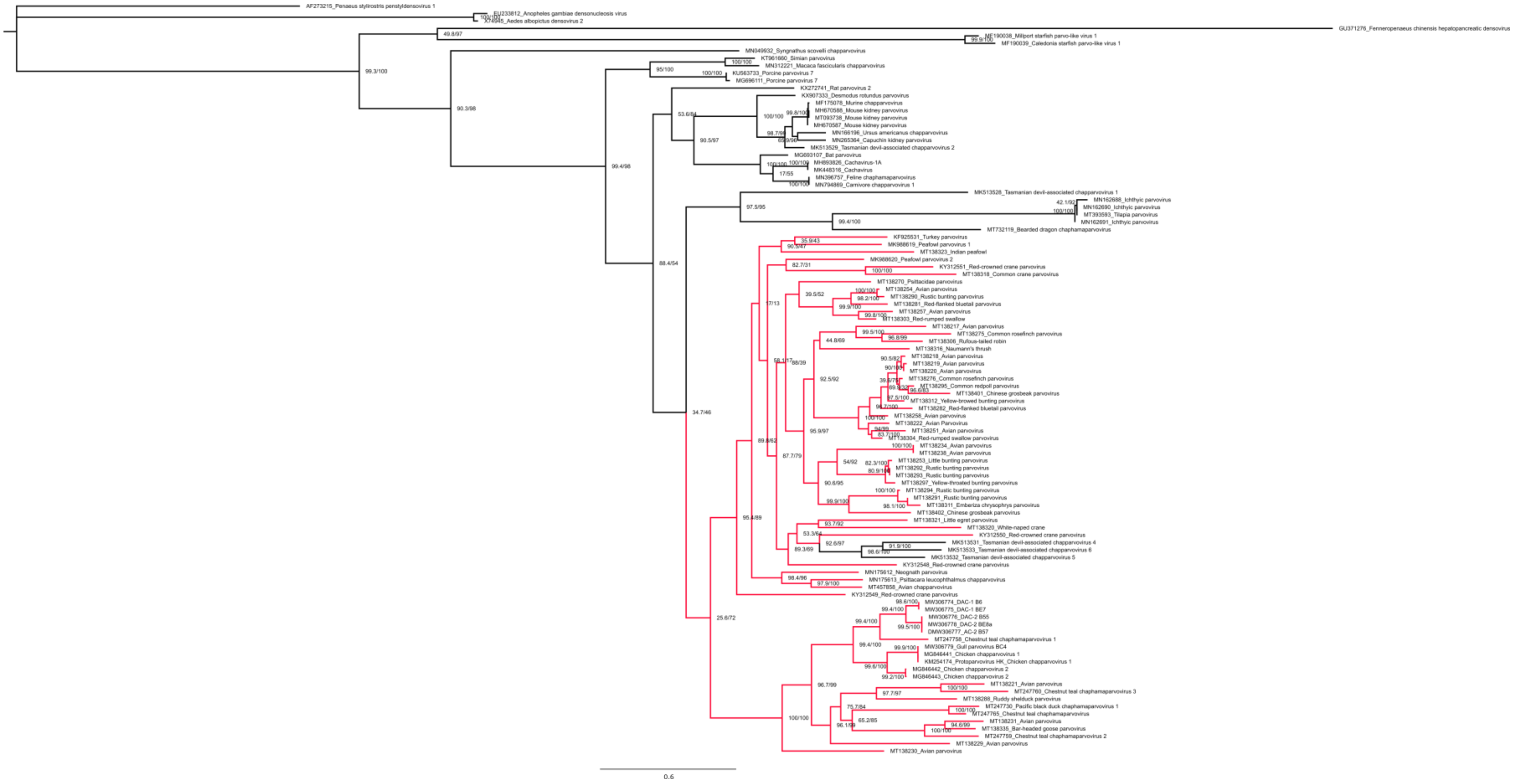

Supplementary Figure S2. Phylogenetic analyses of the partially sequenced duck-associated chapparvoviruses (DAC).
The phylogenetic trees based on nucleotide sequences were built with the maximum-likelihood method based on the GTR+F+I+G4 (top-left), TVM+F+I+G4 (top-right), and TIM+F+G4 (bottom) models with IQ-Tree. The outcome of the SH-aLRT and bootstrap test are shown for all nodes. The trees show the placements of strains B56b (1068 nt), BE8b and B56a (880 nt), and BF2 (134 nt). The viruses identified in this study are labelled with a colored full circle (red for those found in ducks and black for the one found in a gull), while viruses found by others in ducks (genus Anas) are indicated by an empty red circle.

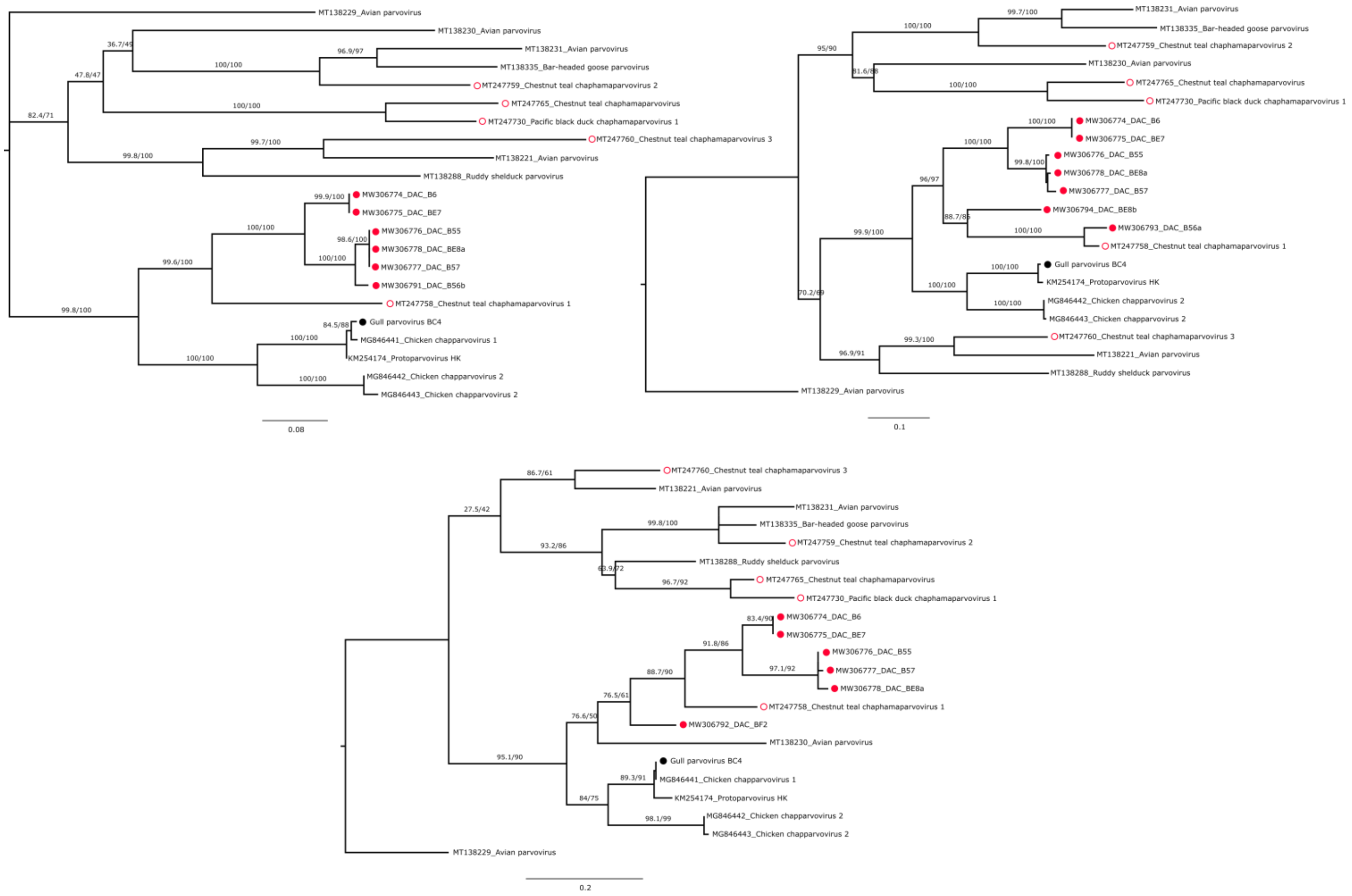

Supplementary Figure S3. Phylogenetic analysis of the duck-associated ambidensovirus (DAAD) within the *Densovirinae*.

The phylogenetic tree based on 117 full NS1 protein sequences was built with the maximum-likelihood method based on the LG+F+R6 model with IQ-Tree. The outcome of the SH-aLRT and bootstrap test are shown for main nodes. The branches of the tree are color-coded based on the taxonomy and red represents putative members of the genus *Protoambidensovirus*.

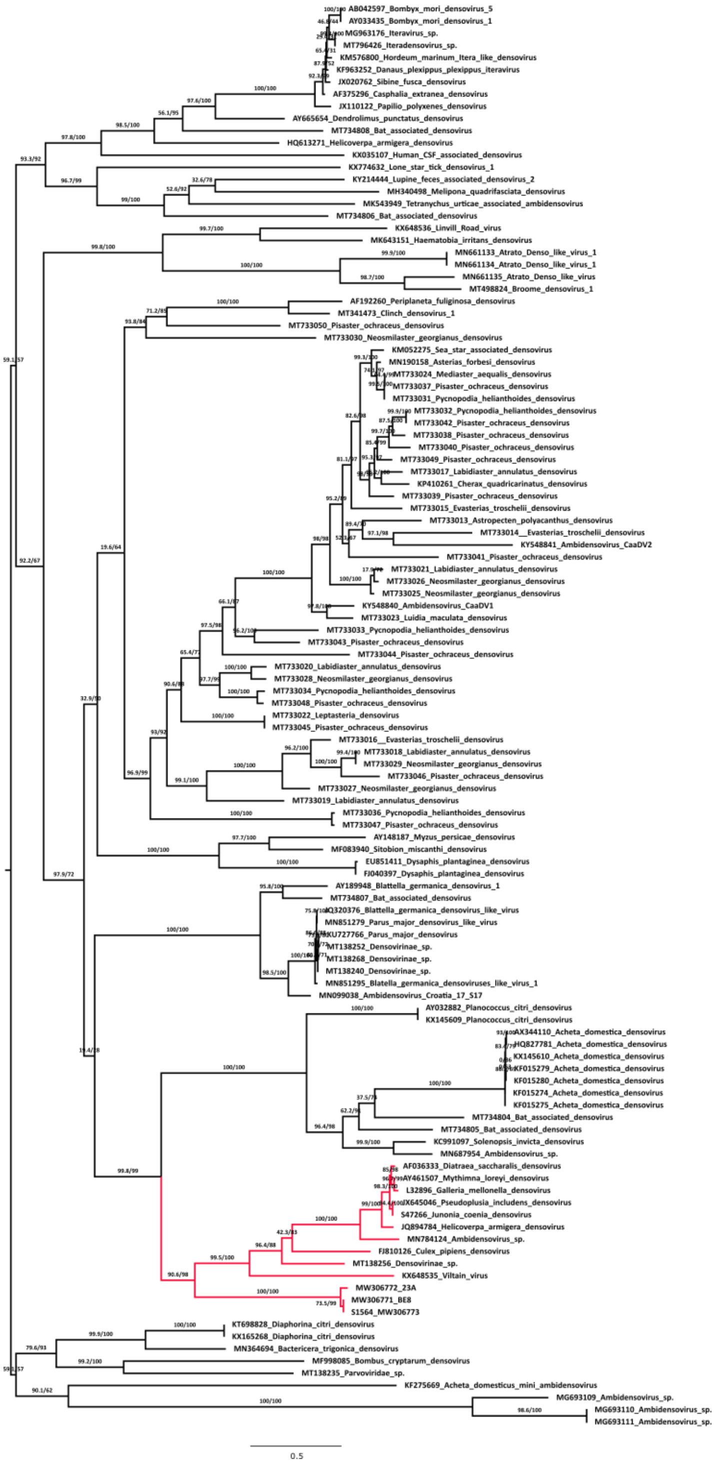

Supplementary Figure S4. Similarity plot between duck-associated ambidensoviruses (DAAD) identified in this study.

The similarity plot was generated with Simplot using strain BE8 as query. The identity between the two strains s1564 and 23A and the query (Y axis) across the genome (X axis) is shown and parameters used for the analysis are indicated at the bottom.

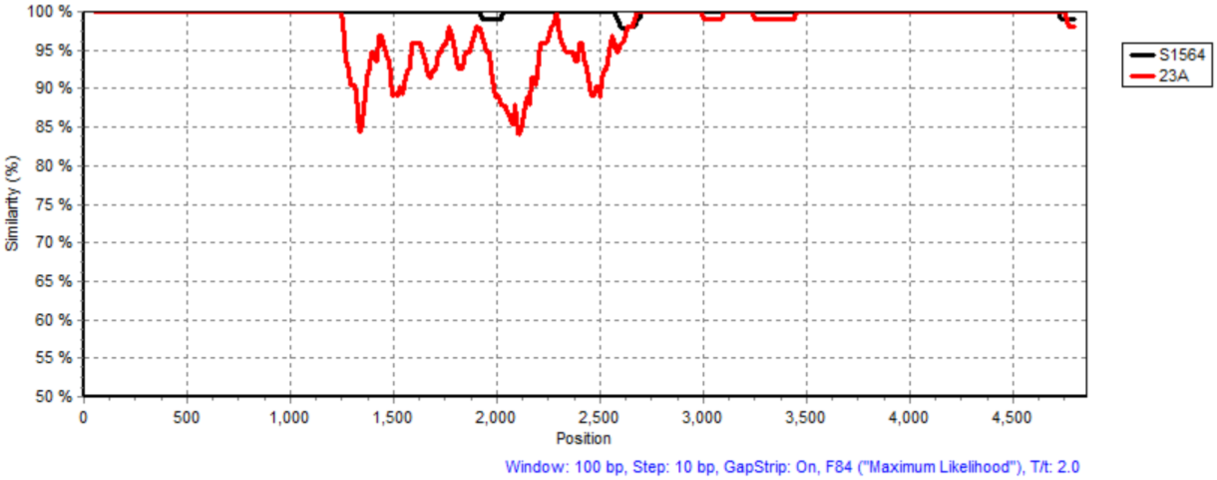

Supplement: Supplementary file 1 [file viruses-13-00193-s001.pdf]
